# Nuclear Import of HIV-1 Complex Relies on a Surprise Player: tRNA

**DOI:** 10.1371/journal.pbio.0040364

**Published:** 2006-10-03

**Authors:** Richard Robinson

To complete its life cycle, it is not enough for HIV-1, the virus responsible for AIDS, to get inside a cell. It must also integrate into the host cell’s DNA. In a dividing cell, the virus can get access to the cell’s DNA when the nuclear envelope dissolves during mitosis. But in cells that do not divide, such as the macrophages of the immune system, the virus must somehow insert its “reverse transcription complex” (RTC) into the nucleus. The RTC contains the virus’s RNA genetic material, as well as proteins that will copy it into DNA and integrate the copy into the host genome.

That it does get into the nucleus has been known for some time, but the details of how it does so are still mysterious. In a new study, Lyubov Zaitseva, Richard Myers, and Ariberto Fassati reveal a surprising key player—the host cell’s own transfer RNA (tRNA). During protein synthesis, tRNA matches amino acids to messenger RNA.

Attempts have been made to study these details before by systematically mutating the virus and identifying genes and sequences critical for successful nuclear import. But the results have not been clear-cut, since on the one hand, mutations often alter more than one character of the virus, and on the other, there may be multiple pathways for nuclear import, not all of which are disrupted by single mutations.

To circumvent these problems, the authors took a different approach. They isolated RTCs and labeled them with a fluorescent tag that allows the authors to track the complexes. They then introduced the RTCs into cells whose cytoplasm had been removed, leaving only the cell’s cytoskeleton and nucleus in place. Using a high-speed centrifuge, they separated cytoplasm from cultured cells into fractions and tested each fraction for its ability to promote nuclear import of the RTCs in the gutted cells. As expected, when they added in the fraction containing several known nuclear transport factors, the RTCs could enter the nucleus. But even after these known factors were removed from the fraction, it still promoted nuclear import, indicating the presence of other, unidentified players.

By further purifying and characterizing the active fraction, the authors showed that the molecules responsible were small RNAs, whose sequences were very close to standard human tRNAs. The small RNAs differed from the standard variety in several ways, possibly reflecting defects in their synthesis. In particular, they lacked a terminal triplet of nucleotides, CCA, which normally binds an amino acid, and whose absence appeared to be critical for the import process; full-length normal tRNAs promoted import only poorly, and when the authors added CCA ends to synthesized versions of their newly identified tRNAs, the import ability of the tRNAs fell. The authors also found that these damaged tRNAs were incorporated into budding virus particles—which may use tRNAs for the next round of infection—and that this activity was promoted by one of the virus’s proteins. Finally, and most remarkably, they found that nuclear import of damaged tRNAs did not require RTC but did require cellular energy, suggesting it is a normal cellular process, not just a consequence of HIV-1 infection.

**Figure:**
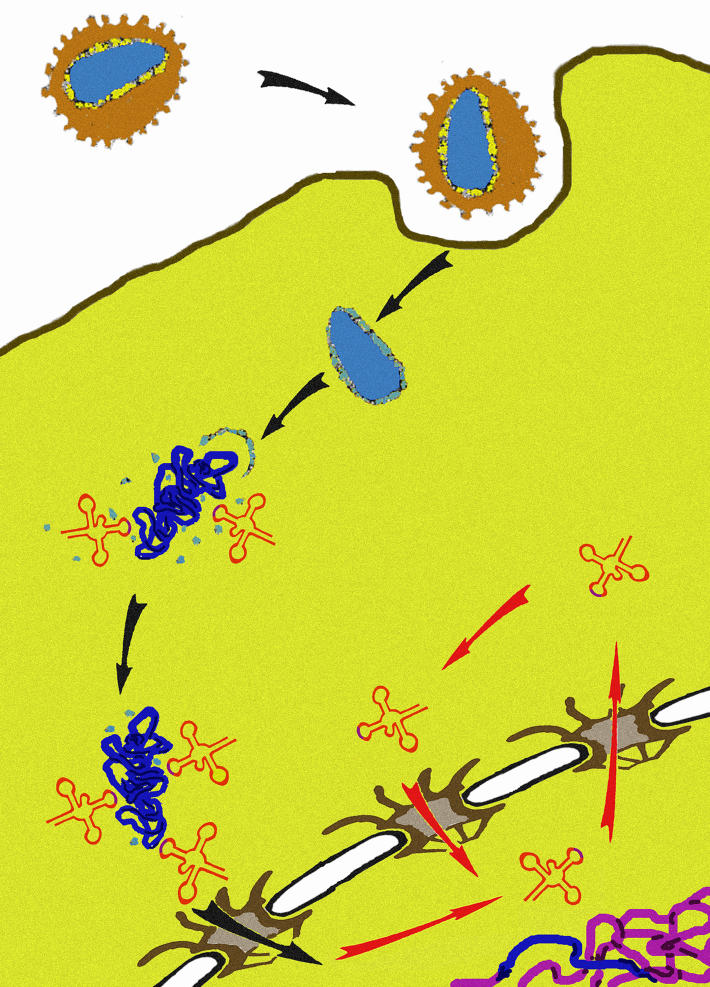
HIV-1 exploits retrograde transport of tRNAs in human cells to promote nuclear import of its reverse transcription complex. (Image: Lyubov Zaitseva)

The discovery of tRNA-mediated import of RTCs helps to further characterize the life cycle of this important and grimly fascinating virus. But perhaps equally interesting is the discovery that nuclear import of tRNA occurs at all and that it appears to be a standard part of the cell’s repertoire. A similar transport activity has recently been seen in yeast, and some evidence suggests it may be a mechanism for sequestration and repair of damaged tRNAs; by removing damaged tRNAs from the cytoplasm, protein synthesis may continue without interference. But it is also possible that undamaged tRNAs enter (and quickly re-exit) the nucleus; further study will be needed to explore these possibilities.

